# Evaluation of body mass index in patients with dipper and non-dipper hypertension

**DOI:** 10.3389/fcvm.2025.1689811

**Published:** 2025-10-20

**Authors:** Tolga Memioğlu, Mehmet İnanır, Kıvanç Argana, Salih Vahit Kiriş, İbrahim Güven, Murat Dıramalı, Kenan Toprak, Mehmet Özyaşar

**Affiliations:** ^1^ Department of Cardiology, Faculty of Medicine, Abant Izzet Baysal University, Bolu, Türkiye; ^2^ Department of Cardiology, Faculty of Medicine, Harran University, Şanlıurfa, Türkiye; ^3^ Department of Cardiology, Konya City Hospital, Konya, Türkiye

**Keywords:** non-dipping blood pressure, body mass index, hypertension, circadian rhythm, obesity

## Abstract

**Background:**

Circadian variation in blood pressure, particularly the distinction between dipper and non-dipper profiles, plays a critical role in cardiovascular risk stratification. Although recent studies suggest that obesity may contribute to non-dipping patterns, the relationship remains controversial.

**Objective:**

To compare body mass index (BMI) between dipper and non-dipper hypertensive patients and to evaluate whether BMI can serve as a marker of non-dipping status.

**Methods:**

This retrospective observational study analyzed ambulatory blood pressure monitoring (ABPM) records of 200 patients (100 dippers and 100 non-dippers) who underwent 24-h ABPM using a validated Holter device. BMI values were calculated and compared between groups.

**Results:**

The distribution of sex did not differ significantly between dipper and non-dipper patients (*p* = 0.571). The mean BMI was significantly higher in the non-dipper group compared with the dipper group (30.75 ± 5.42 vs. 28.07 ± 5.09 kg/m^2^, *p* = 0.002). Non-dipper hypertensive patients demonstrated significantly higher BMI levels than dipper patients.

**Conclusions:**

These findings support the hypothesis that obesity may impair nocturnal blood pressure regulation and suggest that BMI could serve as a simple marker of non-dipping status. This indicates a potential association between increased BMI and a non-dipping blood pressure profile. Due to its retrospective design, this study cannot establish causality.

## 1 Introduction

Hypertension is a highly prevalent and modifiable risk factor for cardiovascular disease (CVD), affecting an estimated 1.28 billion adults globally. It remains a leading contributor to morbidity and mortality related to heart disease and stroke (1). Blood pressure (BP) follows a circadian rhythm, typically decreasing by 10%–20% during nighttime sleep compared to daytime values. This physiological variation allows hypertensive patients to be classified as “dippers” (normal nocturnal BP decline) or “non-dippers” (nocturnal BP decline <10%) based on 24-h ambulatory blood pressure monitoring (ABPM) (2, 3). Importantly, the non-dipping pattern has been identified as an independent risk factor for cardiovascular events, target organ damage, and all-cause mortality (4). In addition to dippers and non-dippers, other circadian blood pressure profiles such as risers (characterized by a paradoxical nocturnal increase compared to daytime BP) and extreme dippers (>20% nocturnal decline) have also been described. Both subgroups have been linked to increased cardiovascular risk (3, 5). Nevertheless, the present study focused on the dipper vs. non-dipper classification, as it remains the most widely used categorization in clinical practice and is emphasized in current hypertension guidelines. However, these subgroups were not analyzed in the present study because the primary focus was on the dipper vs. non-dipper classification, which remains the most widely used categorization in clinical practice. The mechanisms underlying the non-dipping pattern are multifactorial and include autonomic imbalance, sympathetic overactivity, impaired baroreflex sensitivity, and altered sodium handling (2, 3). In recent years, several studies have emphasized the relationship between non-dipping status and metabolic factors such as obesity, insulin resistance, and obstructive sleep apnea (6). Among these, obesity—commonly assessed using body mass index (BMI)—has emerged as a particularly important determinant of circadian BP variability. Papassotiriou et al. further supported this association, highlighting the contribution of obesity to non-dipping patterns (7). Obesity contributes to the development of hypertension through mechanisms such as sympathetic nervous system activation, renin-angiotensin-aldosterone system (RAAS) stimulation, and chronic low-grade inflammation, all of which may interfere with nocturnal BP dipping (6). Given the increasing global burden of both hypertension and obesity, a better understanding of their interaction, particularly in the context of circadian BP patterns, is essential. Identifying whether elevated BMI is associated with a non-dipping profile may provide valuable insight for cardiovascular risk stratification and guide individualized antihypertensive therapy. Therefore, this study aimed to compare BMI values between dipper and non-dipper hypertensive patients to explore the role of BMI in BP circadian variation.

## 2 Methods

### 2.1 Study design and population

This study adhered to the ethical principles outlined in the Declaration of Helsinki (1975) and its subsequent amendments. Ethical approval was obtained from the Bolu Abant Izzet Baysal University Non-Interventional Clinical Research Ethics Committee on January 21, 2025 (decision number: 2025/24). Informed consent was not required due to the retrospective nature of the study. A total of 200 adult patients with a confirmed diagnosis of essential hypertension who had undergone 24-h ambulatory blood pressure monitoring (ABPM) using a validated Holter device (IEM GmbH, Aachen, Germany, Mobil-O-Graph NG) were retrospectively included. Hematological parameters, including hemoglobin, hematocrit, white blood cell (WBC) count, platelet count, red cell distribution width (RDW), and differential leukocyte counts (neutrophils, lymphocytes, monocytes, eosinophils, and basophils) were measured using a Sysmex XN-1000 automated hematology analyzer (Sysmex Corporation, Kobe, Japan). Biochemical parameters were measured using an Abbott Architect c16000 clinical chemistry analyzer (Abbott Laboratories, Illinois, USA). Hemoglobin A1c (HbA1c) levels were measured using a Lifotronic H9 HbA1c analyzer (Lifotronic Technology Co., Ltd., Shenzhen, China) and high-performance liquid chromatography (HPLC).

The age range of the participants was 18–80 years. Based on nocturnal BP decline, patients were categorized into two groups: non-dippers (*n* = 100), defined as those with <10% nighttime systolic BP reduction, and dippers (*n* = 100), defined as those with ≥10% nocturnal systolic BP reduction, in accordance with current hypertension guidelines.

### 2.2 Inclusion and exclusion criteria

Eligible participants were adults (≥18 years) with a confirmed diagnosis of essential hypertension who had complete and technically adequate 24-h ABPM recordings suitable for circadian BP analysis. Only patients with ≥70% valid ABPM readings, including at least 20 daytime measurements (≥2 per h) and ≥7 nighttime measurements (≥1 per h), were included in the final analysis.

Both patients who had not previously received any antihypertensive treatment and those on stable antihypertensive therapy for at least 4 weeks prior to enrollment were included. Exclusion criteria were: severe valvular heart disease; advanced heart failure (left ventricular ejection fraction <30%); severe hepatic or renal failure; chronic lung disease; secondary hypertension; age <18 years and >80; and any acute systemic inflammatory or infectious conditions. Patients with significant comorbidities that could interfere with BP regulation (e.g., uncontrolled diabetes mellitus, recent myocardial infarction, or cerebrovascular event within the past 6 months) were also excluded.

### 2.3 Data collection

All data were collected retrospectively from hospital electronic medical records. Demographic characteristics (age, sex), comorbidities, anthropometric measurements, and laboratory results were documented. BMI was calculated as weight (kg) divided by height squared (m^2^).

ABPM was performed using an automated oscillometric device (validated Holter; IEM GmbH, Aachen, Germany, Mobil-O-Graph NG), recording BP every 15 min during the daytime and every 30 min at night. Nighttime BP values were defined as the average of readings taken between 10:00 PM and 6:00 AM, while daytime BP values were defined as the average of readings taken between 6:00 AM and 10:00 PM.

### 2.4 Statistical analysis

Statistical analyses were performed using SPSS (version 22.0; IBM Corp., Chicago, IL, USA). Normality was assessed with the Shapiro–Wilk test. Normally distributed continuous variables are summarized as mean ± standard deviation and compared using two-tailed independent-samples t tests; homogeneity of variances was evaluated by Levene's test (Welch's t reported when violated). Non-normal variables are reported as median (1st–3rd quartiles) and compared using the two-tailed Mann–Whitney *U* test. Categorical variables were compared using the chi-square test (Fisher'sexact test when expected counts <5). Dipping status was derived from ambulatory blood pressure monitoring as the percent nocturnal decline relative to daytime values; a decline <10% defined non-dippers (pre-specified threshold in the literature/guidelines). To identify independent predictors of non-dipping, we fitted a multivariable logistic regression with body mass index (BMI) as the primary predictor and age, HbA1c, ALT, neutrophilcount, and mean platelet volume (MPV) as covariates. We assessed linearity of continuous predictors with the logit (Box–Tidwell procedure) and multicollinearity (varianceinflation factors). Model performance was summarized by the likelihood-ratio test and Nagelkerke *R*^2^; calibration by the Hosmer–Lemeshow test; and discrimination by the area underthe ROC curve (AUC). Influential observations were screened (Mahalanobis distance). For each predictor, we report the regression coefficient (estimate), standard error (SE), odds ratio (OR) with 95% confidence interval (CI), and *p* value. Statistical significance was set at two-tailed *p* < 0.05.

## 3 Results

A total of 200 hypertensive patients were included in the study, with 100 classified as non-dippers and 100 as dippers based on 24-h ambulatory blood pressure monitoring (ABPM).

### 3.1 Demographic and clinical characteristics

As shown in Table 1, the mean age of the non-dipper group was significantly higher than that of the dipper group (*p* = 0.026). The distribution of sex did not differ significantly between the two groups (*p* = 0.571). Body weight was significantly greater in non-dippers compared with dippers (*p* = 0.019), while height was similar between groups for non-dippers (*p* = 0.949).

**Table 1:** Demographic and laboratory characteristics of non-dipper and dipper groups.

Variables	Non-dipper (*n* = 100)	Dipper (*n* = 100)	*p*
Age (Year)	62.95 ± 14.44	58.46 ± 13.84	0.026
Sex (F)	54 (54.0%)	50 (50.0%)	0.571
Weight (kg)	84.45 ± 12.76	79.98 ± 13.93	0.019
Height (m)	1.65 (1.6–1.7)	1.65 (1.6–1.72)	0.949
BMI (kg/m^2^)	30.7 (28.0–33.4)	28.1 (26.0–31.0)	0.002
EF (%)	60 (57–60)	60 (60–65)	0.075
Heart rate (bpm)	74 (68–82.75)	76 (68–84)	0.474
Smoking (Yes)	15 (28.8%)	18 (32.1%)	0.710
DM (Yes)	29 (33.7%)	19 (23.2%)	0.130
Fasting glucose (mg/dl)	108.5 (92.75–125)	98 (91–115)	0.029
Hemoglobin A1c (%)	5.9 (5.525–6.6)	5.7 (5.4–6.05)	0.029
Alanin aminotransferase (U/L)	17 (13–23)	20 (16–29.5)	0.006
Aspartate aminotransferase (U/L)	19 (16–24)	22 (17–25)	0.073
Triglycerides (mg/dl)	125.5 (98.25–200)	129.5 (89.25–191)	0.89
Total cholesterol (mg/dl)	199.5 (163.25–219)	201 (173.75–224.5)	0.372
High-density lipoprotein cholesterol (mg/dl)	46 (38–51)	47 (40–57)	0.199
Low-density lipoprotein cholesterol (mg/dl)	118.5 (97.25–137)	119 (100.25–144.5)	0.682
C-reactive protein (mg/L)	3 (1–6)	2.3 (1–5.9)	0.904
Sodium (mmol/L)	140 (137–142)	139 (138–140)	0.138
Potassium (mmol/L)	4.41 ± 0.45	4.34 ± 0.42	0.305
Creatinine (mg/dl)	0.9 (0.78–1.03)	0.89 (0.78–1.01)	0.862
Glomerular filtration rate	81 (67–95)	86.5 (72.25–98.75)	0.129
Urea (mg/dl)	34 (24–43)	30 (24–39)	0.299
Uric acid (mg/dl)	5.6 (4.875–7.1)	6.05 (4.475–6.725)	0.588
Thyroid-stimulating hormone (uIU/ml)	1.2 (0.818–2.075)	1.29 (0.89–2.1)	0.398
Hemoglobin (g/dl)	13.65 ± 1.66	13.92 ± 1.49	0.247
Hematocrit (%)	41.33 ± 4.41	41.56 ± 4.35	0.722
Mean corpuscular volume (fL)	85.8 (82–89.5)	87 (83.3–90)	0.084
Mean platelet volume (fL)	9.67 ± 1.58	9.14 ± 1.50	0.022
Red cell distribution width (%)	14.1 (13.3–15.6)	13.6 (12.95–15.4)	0.001
White blood cell count (10³/μl)	7.4 (6.3–8.7)	6.9 (5.645–7.9)	0.037
Neutrophil (10³/μl)	4.5 (3.5–5.54)	3.7 (2.97–4.6)	0.003
Lymphocyte (10³/μl)	2 (1.6–2.5)	2.13 (1.7–2.6)	0.176
Eosinophil (10³/μl)	0.16 (0.1–0.22)	0.15 (0.1–0.2)	0.687
Basophil (10³/μl)	0.04 (0.02–0.07)	0.03 (0–0.065)	0.052
Monocyte (10³/μl)	0.58 (0.46–0.7)	0.6 (0.43–0.7)	0.841
Platelet count (10³/μl)	256 (218–315)	259 (229–300)	0.768
Plateletcrit (%)	0.25 (0.19–0.3)	0.24 (0.2–0.28)	0.381
Platelet distribution width (%)	16 (11.9–17.2)	16.7 (14.25–17.05)	0.173

Data are presented as mean ± standard deviation, median (1st–3rd quartile values), or number (percent, %). Student's *t*-test, Mann–Whitney *U* test, and Pearson's chi-square test were used, as appropriate.

**Figure 1:**
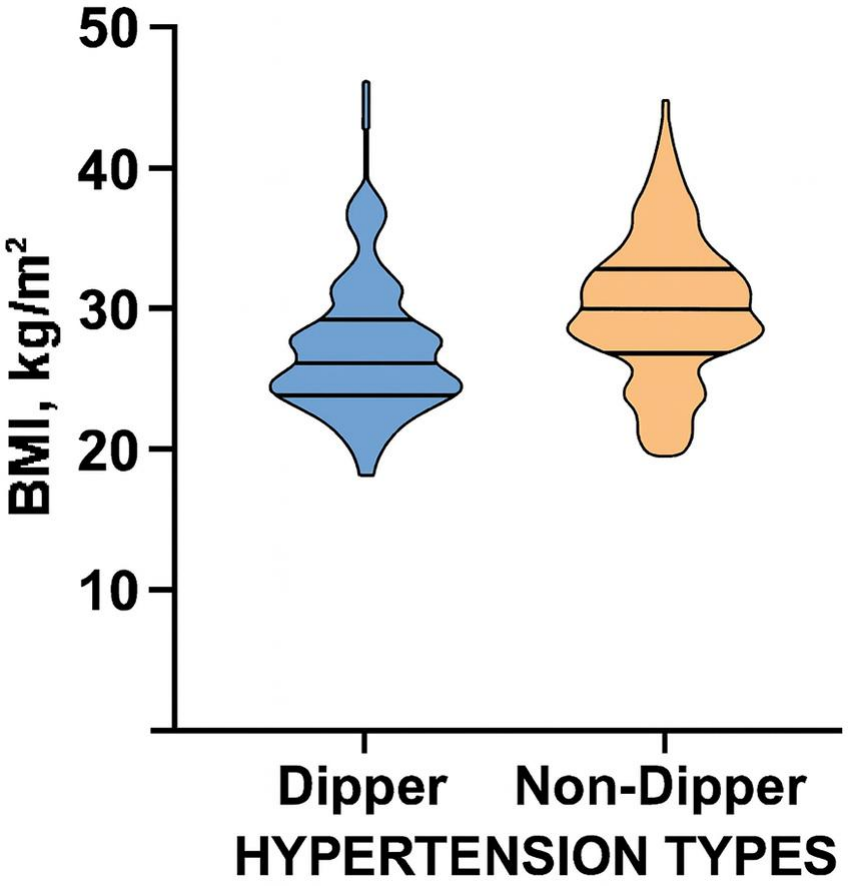
Violin plot illustrating the comparison of body mass index (BMI) between dipper and non-dipper patients.

BMI, the primary variable of interest, was notably higher in non-dippers: the median BMI was in non-dippers compared with in dippers, a statistically significant difference (*p* = 0.002) (Figure 1).

Other variables such as heart rate, smoking status, and prevalence of diabetes mellitus did not differ significantly between the groups. For example, smoking was reported in 28.8% of non-dippers and 32.1% of dippers (*p* = 0.710). Similarly, the prevalence of diabetes mellitus was higher in non-dippers, although this difference did not reach statistical significance (*p* = 0.130).

### 3.2 Laboratory findings

Table 1 summarizes the laboratory findings of the study population.

With respect to liver enzymes, ALT levels were significantly lower in the non-dipper group compared with the dipper group (*p* = 0.006), while AST levels showed a non-significant trend toward lower values in non-dippers (*p* = 0.073). Regarding glucose metabolism, fasting glucose levels were higher in the non-dipper group than in the dipper group (*p* = 0.029), and HbA1c values were also higher in the non-dipper group (*p* = 0.029). Inflammatory markers were also elevated in the non-dipper group. WBC count was higher in the non-dipper group than in the dipper group (*p* = 0.037), and neutrophil counts were also higher in the non-dipper group (*p* = 0.003). For platelet indices, mean platelet volume (MPV) was significantly higher in non-dippers compared with dippers (*p* = 0.022). No significant differences were observed in platelet distribution width (PDW) or platelet count (PLT) (*p* > 0.05 for both). Lipid profile parameters (LDL, HDL, total cholesterol, triglycerides) and renal function markers (creatinine, eGFR, urea) were comparable between the groups (*p* > 0.05 for all). Similarly, other hematological indices, including hemoglobin, hematocrit, eosinophil count, and monocyte count, did not differ significantly (*p* > 0.05 for all). However, RDW was significantly higher in non-dippers compared with dippers (*p* = 0.001).

A multivariate logistic regression analysis was conducted to identify independent predictors of non-dipper status (Table 2). The model included age, BMI, HbA1c, ALT, neutrophil count, and MPV. Among these, only BMI was significantly associated with non-dipper status, indicating that higher BMI increased the likelihood of being a non-dipper (*p* = 0.035). Age, HbA1c, neutrophil count, ALT, and MPV were not significant predictors (*p* > 0.05 for all).

**Table 2:** Predictors for non-dipping blood pressure pattern.

Variables	Estimate ± SE	OR (95% CI)	*p* *
Age	0.022 ± 0.015	1.022 (0.993–1.052)	0.136
Body mass index (kg/m^2^)	0.082 ± 0.039	1.085 (1.006–1.171)	0.035
Alanine aminotransferase (U/L)	−0.036 ± 0.019	0.964 (0.929–1.001)	0.059
Hemoglobin A1c (%)	0.091 ± 0.172	1.095 (0.783–1.533)	0.596
Neutrophils (×10³/μl)	0.148 ± 0.119	1.16 (0.919–1.464)	0.212
Mean platelet volume (fL)	0.199 ± 0.123	1.22 (0.96–1.552)	0.104

OR, odds ratio; CI, confidence interval; SE, standard error.

* *p* value of multivariable logistic regression analysis.

The full model demonstrated a significant improvement over the null model (Δ*χ*^2^ = 23.339, df = 6, *p* < 0.001). Pseudo-*R*^2^ values indicated acceptable model performance (Nagelkerke *R*^2^ = 0.190). Non-dippers exhibited significantly higher HbA1c levels than dippers, suggesting impaired glycemic control (*p* = 0.029). MPV was also significantly higher among non-dippers compared with dippers (*p* = 0.022).

## 4 Discussion

### 4.1 Main finding

This study aimed to explore the relationship between circadian BP variation and clinical-metabolic parameters, with a specific focus on BMI in hypertensive individuals. The findings revealed that patients with a non-dipper BP profile exhibited a more adverse cardiometabolic and inflammatory profile compared with dippers. Non-dippers group were significantly older, had higher BMI and body weight, and demonstrated elevated markers of impaired glucose metabolism and low-grade inflammation, including increased fasting glucose, HbA1c, WBC, and neutrophil counts. Furthermore, MPV, a potential marker of platelet activation and cardiovascular risk, was also higher among non-dippers in univariate comparisons; however, it did not retain independent predictive value in multivariate analysis. Despite several variables differing between the groups, BMI emerged as the only independent predictor of non-dipping status. These findings suggest a strong association between excess body weight and the disruption of normal nocturnal blood pressure decline, and contributes to the literature supporting the association between obesity and impaired circadian cardiovascular regulation.

What is particularly novel in our study is the demonstration that, after adjustment for multiple potential confounders, BMI remained the only independent predictor of non-dipping status. While prior studies have linked obesity and non-dipping patterns, our results emphasize that BMI uniquely retained predictive value even when glycemic, inflammatory, and hematologic markers were considered. This underlines the potential role of BMI not merely as a correlate but as a central determinant of circadian blood pressure dysregulation and highlights a distinct contribution of our findings to the existing literature. However, it should be noted that the regression model explained only a modest proportion of the variance (Nagelkerke *R*^2^ = 0.19), indicating that while BMI is important, additional factors likely contribute to the non-dipping phenotype. Nevertheless, the suggestion that BMI alone could serve as a clinical marker of non-dipping status should be interpreted with caution, and prospective studies are needed to validate this finding.

### 4.2 Comparison with prior studies

Several studies have investigated the relationship between BMI and dipping status. For example, Papassotiriou et al. demonstrated that obese hypertensive adults exhibit up to a 42% higher likelihood of non-dipping compared with those of normal weight (7). Tałałaj et al. found significantly higher BMI values among non-dipper hypertensive patients, supporting the hypothesis that increased adiposity may be linked to impaired blood pressure rhythm (8). Similarly, our results showed a statistically significant elevation in BMI and body weight among non-dippers, reinforcing the notion that obesity is a key factor in circadian BP dysregulation. In contrast, Moczulska et al. did not observe a significant difference in BMI between dipper and non-dipper groups (9). This discrepancy may suggest that obesity alone does not fully account for dipping status in all hypertensive populations and that other metabolic or autonomic factors may also be involved.

The higher BMI observed in non-dippers may be attributable to various pathophysiological mechanisms. Hall et al. reported that obesity promotes sympathetic nervous system activation and RAAS upregulation, both of which interfere with normal nocturnal blood pressure reduction (6). In addition, obstructive sleep apnea (OSA)—a condition more prevalent among obese individuals—has been associated with blunted nocturnal BP decline. This occurs through mechanisms such as intermittent hypoxia, frequent nighttime arousals, and increased sympathetic drive, as described by Somers et al. (10). Drager et al. similarly emphasized the role of OSA in disrupting cardiovascular autonomic regulation during sleep, further contributing to non-dipping patterns (11). A recent review highlighted that up to 84% of untreated moderate-to-severe OSA patients exhibit a non-dipping pattern, mediated by intermittent hypoxia, elevated sympathetic tone, and RAAS activation (12). Although OSA status was not assessed in our study, the observed constellation of higher BMI, elevated glucose parameters, and increased inflammatory markers in non-dippers may serve as indirect indicators of underlying sleep-disordered breathing. These findings suggest that clinicians should consider the possibility of unrecognized OSA in obese hypertensive patients with non-dipping BP profiles.

In this study, higher fasting glucose and HbA1c levels in non-dippers suggested impaired glycemic control. Sun et al. reported that non-dipping status is more prevalent among individuals with insulin resistance or type 2 diabetes mellitus (13). Furthermore, Luo et al. demonstrated that non-dipping blood pressure profiles in hypertensive patients with OSA are independently associated with the development of new-onset diabetes (14). In our study, similar to the findings of Ukkola et al., non-dippers had significantly higher fasting glucose levels and a more adverse metabolic profile, further supporting the association between impaired glucose regulation and the non-dipping pattern (15). Our results add to this narrative by demonstrating significant differences in glycemic and inflammatory markers, suggesting broader metabolic involvement in non-dipping physiology.

Hotamisligil discussed how chronic low-grade inflammation in obesity contributes to endothelial dysfunction and metabolic dysregulation (16). Our results support this model, as WBC and neutrophil counts were significantly elevated in non-dippers, indicating systemic inflammatory activation.

Chotruangnapa et al. demonstrated that the neutrophil-to-lymphocyte ratio (NLR)—a known inflammatory marker—is significantly elevated in non-dipper hypertensive patients. Their case–control study also identified age and clinical parameters such as metabolic syndrome as strong independent predictors of non-dipping status in treated individuals (17). Similarly, Kim et al. reported that epicardial fat thickness and NLR are predictive of non-dipping blood pressure patterns, suggesting that visceral adiposity may mediate inflammation-driven autonomic dysregulation (18). In our study, higher MPV among non-dippers may reflect increased platelet activation, which could be part of the inflammatory milieu contributing to altered BP patterns.

Mohawk et al. described how disruptions in central and peripheral circadian clocks—often seen in obesity and metabolic syndrome—can impair vascular function and BP regulation (19). While circadian rhythm markers were not directly assessed in our study, the observed clinical and laboratory profile of non-dippers implies potential misalignment in chronobiological regulation.

In a large prospective study, Hermida et al. identified BMI as a significant independent predictor of circadian blood pressure variation, reporting that higher BMI was strongly associated with an increased prevalence of non-dipping blood pressure patterns in hypertensive patients (20). These findings closely mirror our results that BMI is independently associated with non-dipping status.

Findings from the Ohasama Study indicate that both general and central obesity—reflected by higher BMI and waist-to-hip ratio—are significantly associated with masked hypertension, highlighting the importance of fat distribution in cardiovascular risk even in the absence of elevated clinic BP (21). Our study adds to this evidence by showing that, although we did not directly measure waist-to-hip ratio or visceral fat through imaging, BMI alone remained a statistically significant predictor of non-dipping, even after adjusting for multiple confounders. This reinforces the notion that total body adiposity, and possibly central fat accumulation, plays a key role in circadian BP regulation. In which non-dipper patients, who had significantly higher BMI, also demonstrated elevated markers of sympathetic activation such as increased MPV and neutrophil count, both of which may reflect underlying neurohormonal stress contributing to altered morning BP patterns.

### 4.3 Mechanisms

Strengths of our study include the use of 24-h ABPM to classify dipper and non-dipper phenotypes and the inclusion of a broad range of clinical and laboratory parameters. Our findings highlight the potential utility of BMI as a simple and accessible clinical indicator for identifying patients at higher cardiovascular risk due to non-dipping status. This relationship may be explained by obesity-related mechanisms such as sympathetic nervous system activation, RAAS upregulation, chronic low-grade inflammation, and the high prevalence of obstructive sleep apnea, all of which can blunt the normal nocturnal decline in blood pressure. Nevertheless, BMI should not yet be regarded as a stand-alone marker for clinical decision-making. Prospective validation in larger, longitudinal cohorts is required before BMI can be recommended as an independent risk stratification tool in routine practice.

### 4.4 Limitations

Limitations of this study include its retrospective design, the absence of sleep data or polysomnography to confirm obstructive sleep apnea, and the lack of direct measurements of visceral adiposity such as waist circumference or imaging-based fat quantification. In addition, we did not include other key covariates such as lifestyle factors (e.g., diet, physical activity, smoking, and alcohol intake). These variables are known to influence both BMI and circadian blood pressure regulation, and their absence may have limited our ability to fully capture the mechanisms underlying the non-dipping phenotype.

### 4.5 Implications

Future studies incorporating these factors would provide a more comprehensive understanding of the interplay between obesity, metabolic regulation, and circadian BP variation. Although risers and extreme dippers represent clinically relevant subgroups with adverse cardiovascular outcomes, these profiles were not included in our analyses. Our primary aim was to compare dippers and non-dippers, given their clinical relevance and recognition in hypertension guidelines. Future studies should incorporate all circadian BP patterns to provide a more comprehensive understanding of their prognostic implications.

## 5 Conclusion

In conclusion, this study demonstrates a significant association between increased BMI and non-dipping blood pressure patterns among hypertensive patients. Non-dipper individuals not only exhibited higher BMI but also elevated inflammatory and glycemic markers, suggesting that obesity may play a pivotal role in disrupting the physiological nocturnal decline in blood pressure. These findings highlight the potential utility of BMI as a simple and accessible clinical indicator for identifying patients at higher cardiovascular risk due to non-dipping status.

Given the growing global prevalence of both obesity and hypertension, incorporating BMI-based risk stratification into routine hypertensive care may aid in the early identification of high-risk individuals and support personalized treatment strategies. Further prospective studies incorporating direct measures of visceral adiposity, objective sleep quality assessment, and long-term cardiovascular outcomes are warranted to validate and expand upon these findings. Due to its retrospective design, this study is limited in its ability to establish causal inferences, and the observed associations should therefore be interpreted with caution.

## Data Availability

The raw data supporting the conclusions of this article will be made available by the authors, without undue reservation.
